# Effect of D-ribose-L-cysteine on aluminum induced testicular damage in male Sprague-Dawley rats

**DOI:** 10.5935/1518-0557.20170023

**Published:** 2017

**Authors:** Benedict Falana, Opeyemi Adeleke, Mulikat Orenolu, Abraham Osinubi, Adeoye Oyewopo

**Affiliations:** 1 Department of Anatomy, College of Health Sciences, Osun State University, Osogbo, Nigeria; 2 Department of Anatomy, College of Medicine, University of Lagos, Lagos State, Nigeria; 3 Department of Anatomy, College of Health Sciences, University of Ilorin, Ilorin State, Nigeria

**Keywords:** testes, aluminum chloride, D-Ribose and L-Cysteine, sprague-dawley rats

## Abstract

**Objective:**

This study investigated the effects of D-ribose and L-cysteine on aluminum-induced testicular damage in male Sprague-Dawley rats.

**Method:**

A total number of thirty-five (35) adult male Sprague-Dawley rats were divided into four groups (AD). Group A (comprised five (5) rats) was designated the Control Group that received Physiological Saline; while groups B, C, and D (comprised ten (10) rats) were given 75 mg/kg, 150 mg/kg and 300 mg/kg of body weight of aluminum chloride respectively for 39 days. At day 40, the aluminum-treated groups were subdivided into sub-groups (B1, C1, D1) comprising of five (5) rats each, and 30 mg/kg body weight of Riboceine were administered for twenty (20) days. Groups B, C and D remained on the normal dosage of aluminum chloride for three more weeks (59 days).

**Results:**

Andrological parameters (Sperm count, motility, morphology and testosterone) in the aluminum-treated Groups B and C showed no significant difference in their mean values when compared with their control counterparts, whereas there was a significant reduction in the andrological parameters in Group D rats when compared with the Control animals. Histoarchitecture of the testes "stain with H&E" of Group A, B and C rats appeared normal while Group D rats showed testicular damages with several abnormal seminiferous tubules with incomplete maturation of germinal cell layers and absence of spermatozoa in their lumen; Leydig cells appear hyperplastic. Group B1, C1 and D1 andrological and histological parameters appeared normal.

**Conclusion:**

Riboceine treatment significantly attenuates aluminum-induced testicular toxicity in male Sprague-Dawley in rats.

## INTRODUCTION

Mammals', especially human's exposure to metals cannot be overlook. Metals are ubiquitous and humans can be exposed to it via pollution in the air, water, soil and food, their wide use in industry and long-term persistence in the environment. Among these metals is aluminum (Al). Aluminum is the most widely distributed trivalent cation, which is usually present in plants, animal tissues and in natural water, in the form of ion. Aluminum prevalence is high when compared to other elements, and its present in the earth crust is more than enough, representing approximately 8% of its total mineral components. Aluminum is present in trace amounts in biological materials and the Agency for Toxic Substances and Disease Registry from the year 2008 reported that aluminum is characterized to have negative effects on general human health (^ATSDR, 2008^). Al occurs naturally in the environment, and it is also released due to anthropogenic activities such as mining and industrial uses, in the production of aluminum metal and other forms of Al compounds. A variety of Al compounds are produced and used for different purposes, such as in water treatment, papermaking, fire retardants, fillers, food additives, pharmaceuticals, colors, cosmetics, Aluminum ware and containers.

Many studies have reported on Aluminum-induced toxic effects in humans or in laboratory animals. Overexposure to aluminum has been reported to have deleterious effects on testicular tissues (^Pizent *et al.*, 2012^). Metals have been shown to affect spermatogenesis in rodents and humans, which can lead to low sperm count, abnormal sperm morphology and poor semen quality (^Pizent *et al.*, 2012^).

Experimental studies have suggested that certain mechanisms might be involved in the male reproductive toxicity of aluminum, and these include impairment of the blood-testis barrier, histological alterations and increased oxidative stress (^Berihu *et al.*, 2015^). Oxidative stress reflects an imbalance between the systemic manifestation of reactive oxygen species and a biological system's ability to readily detoxify the reactive intermediates or to repair the resulting damage. Thus, causing disruptions in normal mechanisms of cellular signaling and cell death (^*Chandra et al., 2015*^). One of the biological systems (antioxidants) that detoxifies reactive oxygen species or that repairs the resulting damages caused by free radicals is Glutathione. Sometimes these free radicals overpower the biological systems, thus the body may need external supplements to complement the production of antioxidants (^*Chandra et al., 2015*^).

Riboceine is one of the synthetic antioxidants that help cells produce glutathione on-demand. The active ingredient of riboceine is D-Ribose-L-Cysteine. Whole glutathione consumption cannot be effective because it would be destroyed in the digestion process before reaching the cell. The ribose component of the Riboceine solves these challenges by effectively protecting and delivering the fragile cysteine molecule, enabling the cells to produce glutathione when the cells need it most. Hence, this study is aimed at investigating the oral administration of Riboceine on Aluminum-induced testicular damage in Sprague-Dawley rats.

## MATERIALS AND METHODS

### Aluminum Chloride and Riboceine Preparation

Aluminum chloride was procured and prepared in the Department of Biochemistry, Osun State University, while Riboceine was obtained from the University of Lagos. The preparation was done by separately dissolving 100 mg of the Aluminum Chloride and Riboceine in 100 ml of distilled water (1ml of the solution contain 1mg of the solvent).

### Experimental Design

A total number of thirty-five (35) adult male Sprague-Dawley rats were divided into four groups (A-D). Group A (comprised five (5) rats) was designated the Control group - receiving saline solution, while groups B, C, and D (comprised ten (10) rats) were given 75 mg/kg, 150 mg/kg and 300 mg/kg body weight of aluminum chloride, respectively, for 39 days. At day 40, the aluminum-treated groups were subdivided into sub-groups (B1, C1, D1) comprised of five (5) rats each and 30 mg/kg body weight of Riboceine were administered for Twenty (20) days. Groups B, C and D remained on the normal dosage of aluminum chloride for three weeks more (59 days). The animals were fed with feed from TopFeeds Ltd. Osogbo, Osun State, Nigeria, with ad libitum access to drinking water. All the experimental procedures were done following the experimental guidelines of the Health Research and Ethics Committee (HREC) of the Osun State University, Osogbo Campus, Osogbo, Osun State.

### Animal Slaughter and Samples Collection

The animals were slaughtered on the sixtieth (60^th^) day of the experiment, being anaesthetized with 0.5ml/kg of ketamine hydrochloride and fixed by transcardial perfusion method using 4% paraformaldehyde as fixative agent. Caudal epididymis was excised from the testes prior to perfusion fixation for sperm analysis and the testes were fixed in Bouin's fluid for histological analysis.

### Sperm count assay

Sperm motility was assessed by the method described by ^Rouge & Bowen, 2002^. The spermatozoa were counted by hemocytometer using the improved Neubauer (Deep 1/10 mm, LABART, Germany) chamber, as described by ^Rouge & Bowen, 2002^.

### Sperm morphology and motility assay

Sperm live/dead ratio and motility was determined using 1% Eosin and 5% Nigrosin in 3% sodium citrate dehydrate solution according to the method described by ^Rouge & Bowen, 2002^.

### Testosterone assay

This was carried out with the use of the Testosterone ELISA Kit obtained from Monobind Inc. Lake forest, CA, U.S.A. The essential reagents used for this assay were biotinylated antibody, enzyme-antigen conjugate and a serum native antigen, upon mixing all these reagents, a competitive reaction set-in between the native antigen and enzyme antigen conjugate for a limited number of antibody binding sites. The amount of testosterone able to bind to the testosterone antiserum will be inversely proportional to the concentration of testosterone in the well. The absorbance in each well at 450nm was read in a microplate reader (using a reference wavelength of 620630nm to minimize well imperfections).

### Histological examination

Routine histological processing using Hematoxylin and Eosin staining method was carried out. The testes were fixed in Boiun's, dehydrated in ascending grades of alcohol, cleared in xylene, infiltrated in molten paraffin wax before finally embedded in molten paraffin wax in order to form the paraffin block. The paraffin block containing the tissue was then sectioned by the rotary microtome at 4µσι thickness. The sections were then floated in water bath at 40^0^c and transferred to a glass slide and stained with hematoxylin and eosin stains. The slides were then viewed under light microscope at ×100 and ×400 magnification and photomicrographs were taken at both magnifications.

### Statistical Analysis

Results obtained from the analysis of andrological parameters (Sperm count, sperm morphology, sperm motility and testosterone concentration), of Sprague-Dawley rats were statistically analyzed, to see the correlation between the results we used the Graphpad prism version 5.01. The results were presented as Mean ± SEM (Standard Error of Mean) with significant level at *p*-value < 0.05, while the histological examination, sperm analysis and serum testosterone level were carefully studied and analyzed to establish any correlation between the groups.

## RESULTS

### Andrological parameters

**Figure 1:**
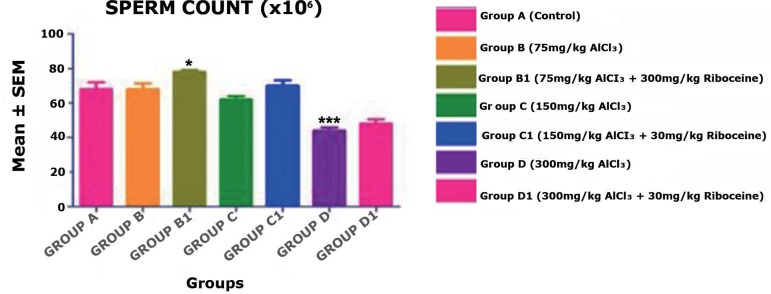
Comparison between the Control and treated groups in terms of sperm counts after administration of Aluminum chloride and D-ribose L-cysteine. Group D, showed a highly significant decrease in sperm count (***p < 0.001); however, Group B1 showed a slightly significant increase in sperm counts (*p < 0.05) when compared with the control Group A rats.

The results depicted on Figure 1 indicate the mean value outcomes of the sperm count, where we found no significant difference in the mean value of sperm count in Groups B, C, C1 and D1 when compared with the Control Group A, but with a slight significant increase (*p* < 0.05) in Group B1, and this may be due to an anti-oxidative effect of D-ribose L-cysteine on the low dose Aluminum administered to Group B rats' testes. Moreover, there was a highly significant decrease (*p* < 0.001) in the sperm count value in Group D rats' given a high dose of Aluminum chloride.

**Figure 2:**
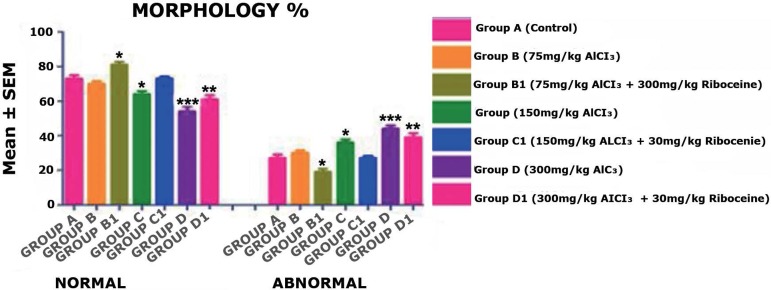
There was no significant difference in the mean value of sperm morphology (Normal and Abnormal) in Group B rats and Group C1 rats, but a slightly significant increase and decrease in Group B1 and C rats, respectively. However, a highly significant decrease was observed in Group D & D1 rats, when compared with the Control Group A rats (*p < 0.05, **p < 0.01, ***p < 0.001).

The result from Figure 2 shows the sperm morphology mean value and it was seen that there was no significant difference in the mean value of sperm morphology (Normal and Abnormal) in Groups B and C1 when compared with the control group A. Moreover, Group B1 rats have slightly significant increase (*p* < 0.05) in the numbers of normal sperm morphology. Group C rats showed slightly significant decreases (*p* < 0.05) in the mean values of normal sperm morphology when compared with the Control Group A, but with the counter administration of D-ribose L-cysteine (Group C1) the number of normal sperm significantly increased. Furthermore, Group D rats showed highly significant decreases (*p* < 0.001) in the mean value of normal sperm morphology when compared with the Control Group A, but with the counter administration of D-ribose L-cysteine (Group D1) the number of normal sperm morphology slightly increased but not up to the mean value of the Control Group A (*p* < 0.01).

**Figure 3:**
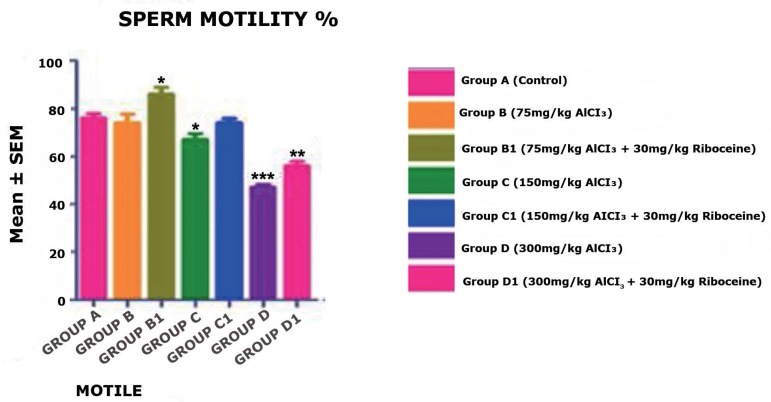
There was no significant difference in the mean value of sperm motility in Group B & C rats, but a slight significant increase and decrease in Group B1 and C rats, respectively. However, a highly significant decrease was found in Group D & D1 rats when compared with the Control Group A rats (*p < 0.05, **p < 0.01, ***p < 0.001).

Moreover, Sperm motility mean values from figure 3 shows no significant difference in the mean value of Groups B and C1 when compared with the control group A. Moreover, Group B1 rats have slightly significant increases (*p* < 0.05) in the numbers of motile sperm. Group C rats showed slightly significant decreases (*p* < 0.05) in the mean value of motile sperm when compared with the Control Group A, but with the counter administration of D-ribose L-cysteine (Group C1) the number of motile sperm significantly increased. Furthermore, Group D rats showed highly significant decreases (*p* < 0.001) in the mean value of motile sperm when compared with the Control Group A; but with the counter administration of D-ribose L-cysteine (Group D1) the number of normal sperm morphology slightly increased, but not up to the mean value of the Control Group A (*p* < 0.01).

**Figure 4:**
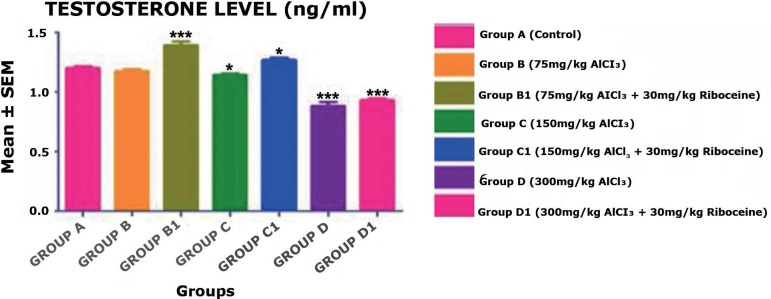
There was no significant difference in the mean value of Serum testosterone level in Group B rats, but a slight significant decrease and increase in Group C & C1 rats, respectively. However, there was a highly significant increase in Group B1 rats, while a highly significant decrease was seen in Group D & D1 rats, when compared with the Control Group A rats (*p < 0.05, *** *p* < 0.001).

Furthermore, serum testosterone levels, as shown in figure 4, revealed that there was no significant difference in the mean value of serum testosterone in Group B rats when compared with the control group A. Moreover, Group B1 rats showed highly significant increases (*p* < 0.001) in the mean value of serum testosterone. Group C rats showed slightly significant decreases (*p* < 0.05) in the mean value of serum testosterone when compared with the Control Group A, but with the counter administration of D-ribose L-cysteine (Group C1) the mean value of serum testosterone significantly increased (*p* < 0.05). Furthermore, Group D rats showed highly significant decreases (*p* < 0.001) in the mean value of serum testosterone when compared with the Control Group A, but with the counter administration of D-ribose L-cysteine (Group D1) the mean value of serum testosterone still significantly decreased (*p* < 0.001) when compare with the Control Group A.

### Histological Examination of the Testes

**Figure 5:**
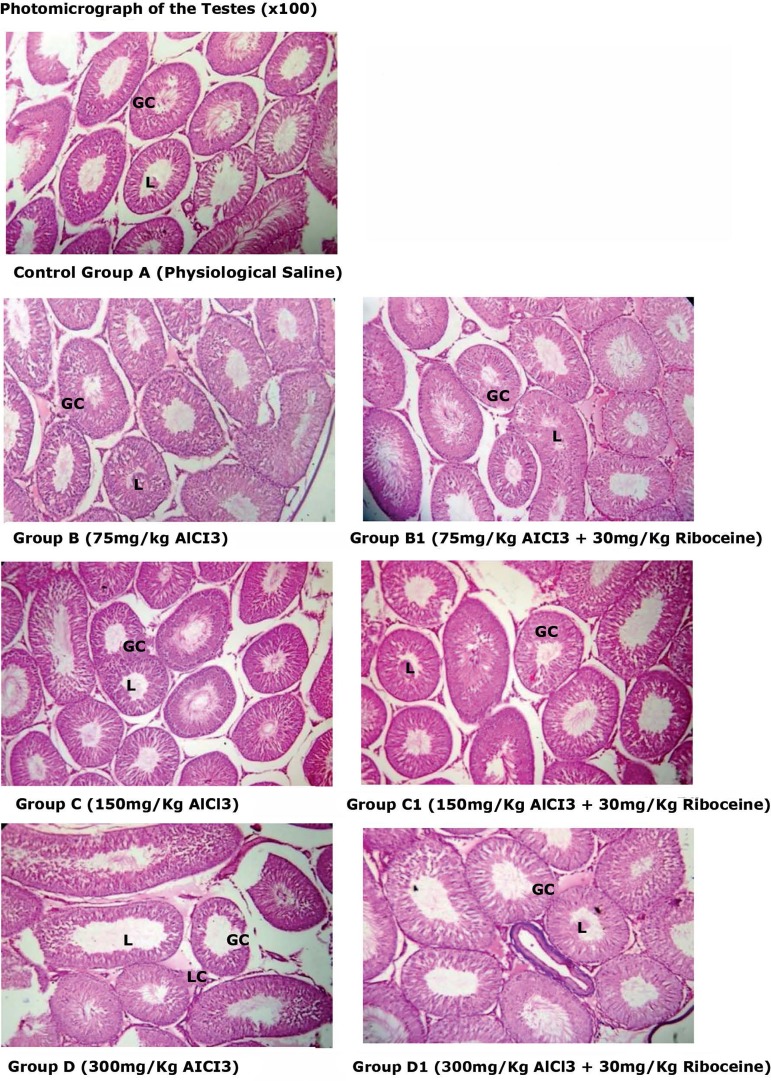
Histoarchitecture of the testes “stained with H&E”. Group A, B and C rats appeared normal, while Group D rats showed testicular damages with several abnormal seminiferous tubules with incomplete maturation of germinal cell layers(GC) and absence of spermatozoa in their lumen (L), leydig cells (LC) appeared hyperplastic. Group B1, C1 and D1 histological parameters appeared normal.

**Figure 6:**
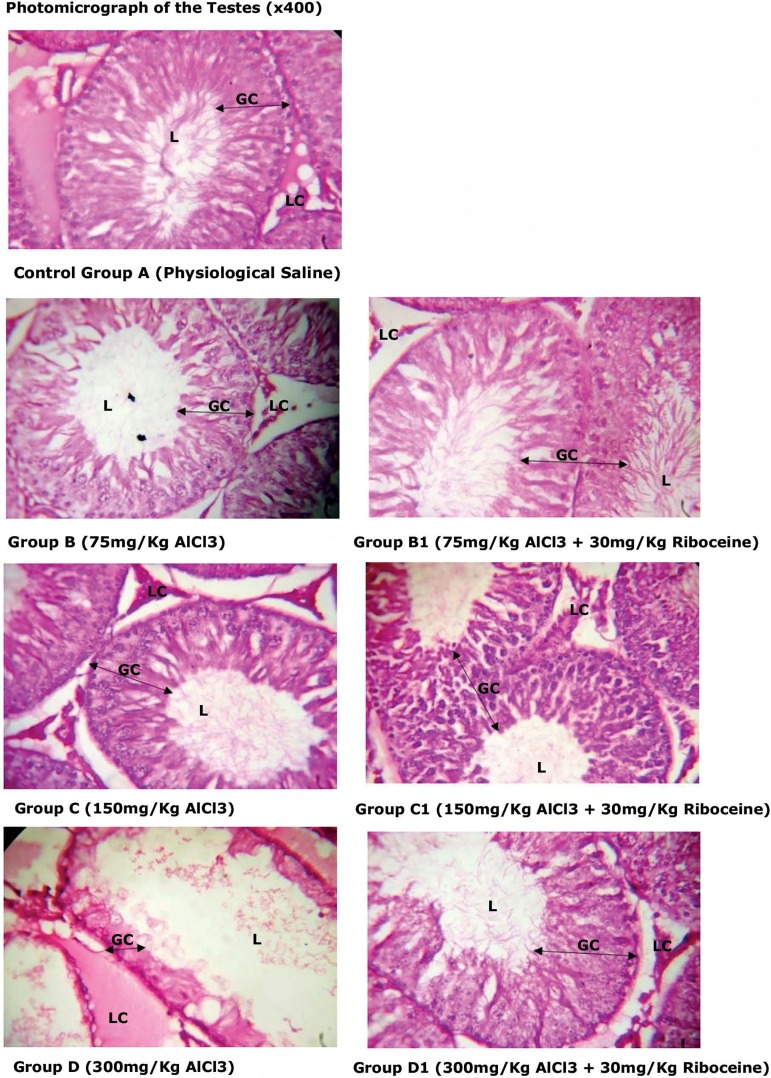
Histoarchitecture of the testes “stained with H&E”. Group A, B and C rats appeared normal, while Group D rats showed testicular damages with several abnormal seminiferous tubules with incomplete maturation of germinal cell layers(GC) and absence of spermatozoa in their lumen (L), leydig cells (LC) appeared hyperplastic. Group B1, C1 and D1 histological parameters appeared normal.

Figures 5 and 6 show the histoarchitecture of the testes "stain with H&E" at x100 and x400 magnification respectively. We found from the slides, that the Group A, B and C rats' testes appeared normal while severe testicular damages with several abnormal seminiferous tubules, incomplete maturation of germinal cell layers(GC), absence of spermatozoa in the lumen (L) and Leydig cells (LC) hyperplasia were seen in Group D rats. Furthermore, Groups B1, C1 and D1 histological parameters appeared normal.

## DISCUSSION

Aluminum chloride at doses of 75 mg/kg and 150 mg/kg of body weight proved normal in all the andrological parameters (sperm count, sperm motility, sperm morphology and serum testosterone), and the testicular histoarchitecture was normal.

AlCl_3_ at a dose of 300 mg/kg body weight was revealed to cause decrease in the andrological parameters, while some abnormalities were observed in the histoarchitecture of the testes. This finding is in line with the study of ^Pizent *et al.* 2012^, in which they reported that aluminum metal affect spermatogenesis in rodents and humans - which can lead to low sperm count, abnormal sperm morphology and poor semen quality. Moreover, ^Berihu *et al.* (2015)^ revealed that the reproductive toxicity induced by aluminum is a result of increased in oxidative stress.

D-ribose and L-cysteine (Riboceine) at a dose of 30 mg/kg for all the groups was found to boost fertility in male Sprague-Dawley rats. D-ribose and L-cysteine at a dose of 30 mg/kg of body weight significantly boosted andrological parameters in group B1 and C1 rats treated with 75mg/kg and 150mg/kg of body weight of Aluminum chloride respectively. Furthermore, D-ribose and L-cysteine at a dose of 30 mg/kg of body weight moderately boosted reproductive parameters damaged by high dose (300 mg/kg body weight) of Aluminum chloride. The increase in andrological parameters seen in the B1, C1 and D1 groups after counter treatment with 30 mg/kg of body weight of D-ribose and L-cysteine may be due to the anti-oxidative protective effects of D-ribose and L-cysteine. The presence of cysteine in the body's cells enables the production of glutathione, while ribose produces cellular energy. It is the interaction between D-ribose and L-cysteine that facilitates the production of glutathione. The Glutathione within the cell protects against destruction from free-radical damage. Providing support to Glutathione, improves ATP production by allowing mitochondria to manufacture ATP, controlling free radicals on an ongoing basis.

### Recommendation

It is therefore recommended that further studies should be carried out to evaluate molecular mechanisms on which D-Ribose and L-Cysteine (Riboceine) work. Moreover, D-Ribose and L-Cysteine (Riboceine) is recommended for any infertility related problems, since it has been revealed from this present study to be a better antioxidant drug because of its beneficiary effects on males. In conclusion, D-Ribose and L-Cysteine (Riboceine) have been shown in this present study to ameliorate the toxic effect of aluminum chloride on the testes, and it will be a good drug for boosting male fertility because of it potent antioxidant property.

## ADDITIONAL INFORMATION

All the experimental procedures were done following the experimental guidelines of the Health Research and Ethics Committee (HREC) of the Osun State University, Osogbo Campus, Osogbo, Osun State.

There was no fund or grant received during this research work.

This manuscript has been read and approved by all participating authors, thus no conflict of interests existed.
